# Supplementary material for: Dynamic transcriptomic profiles of zebrafish gills in response to zinc supplementation
Source: BMC Genomics. 2010 Oct 11;11:553. doi: 10.1186/1471-2164-11-553 (PMC3091702; doi:10.1186/1471-2164-11-553)
Supplement: Additional file 2 — Interactive Direct Interaction Network representing the molecular interactions between zinc, copper, iron, calcium and proteins encoded by transcripts changed by zinc supplementation. Mini web-site containing index.html and hyperlinked pages in subdirectory describing a Direct Interaction Network automatically generated based on curated interactions contained within the proprietary PathwayArchitect database. Ovals represent proteins and the circles symbolize metal ions. Objects are coloured by their abundance in zebrafish at the time-point they were significantly different from the control is a scale from -4 fold (dark green) to +4 fold (dark red). Where significant differences were found at more than one time-point, the colour overlay shows expression at the first instance. Dark blue squares denote 'binding', and light blue squares 'expression'; green squares stand for 'regulation', green diamonds for 'metabolism', and green circles for 'promoter binding'. Arrow heads indicate directionality of the interaction where annotated. All nodes and edges can be further interrogated by selecting the relative area of the image. [file 1471-2164-11-553-S2.zip › PathwayArchitect Zn xs DIN/961654.html]

# REGULATION:

|  |  |
| --- | --- |
| Type | REGULATION |
| Effect | None |


---

|  |  |
| --- | --- |
| Score | 0 |


---

|  |  |
| --- | --- |
| Reference Count | 4 |


---

|  |  |
| --- | --- |
| Mechanism | Unknown |


---

|  |  |
| --- | --- |
| Reference:0 || Sentence | "These results suggest that DMT1, Fpn1, and Heph are involved in the iron uptake process modulated by copper status." |
| PMID | 11842003 |
| Year | 2002 |
| Species | Human |
| Journal | Am J Physiol Gastrointest Liver Physiol |
| RefScore | 0 |
| Source | PArchNLP |
  |
|  |  |


---

|  |  |
| --- | --- |
 Reference:1 || Sentence | "The main results are the following: (i) copper or iron ions, especially in the presence of the reducing agent ascorbate, accelerated the decomposition of cysNO and markedly attenuated the amplitude and duration of the relaxant effect of cysNO; (ii) by contrast, the iron and copper chelators bathophenantroline disulfonic acid (BPDS) and bathocuproine disulfonic acid (BCS) exerted a stabilizing effect on cysNO, prolonged its vasorelaxant effect, and abolished the influence of ascorbate; (iii) in the presence of ascorbate, BPDS displayed a selective inhibitory effect toward the influence of iron ions (but not toward copper ions) on cysNO decomposition and vasorelaxant effect, while BCS prevented the effects of both copper and iron ions; (iv) L-cysteine enhanced stability and prolonged the relaxant effect of cysNO; (v) the process of iron-induced decomposition of cysNO was associated with the formation of EPR-detectable dinitrosyl-iron complexes (DNIC) either with non-thiol- or thiol-containing ligands (depending on the presence of L-cysteine), both of which exhibiting vasorelaxant properties." |
| PMID | 12381416 |
| Year | 2002 |
| Species | Mouse |
|  | Rat |
| Journal | Nitric Oxide |
| RefScore | 1 |
| Source | PArchNLP |
  ||  |  |


---

|  |  |
| --- | --- |
 Reference:2 || Sentence | On the other hand, copper (Cu), manganese (Mn), and iron (Fe) concentrations in the body hair were significantly higher in the ZD rats than in the controls. |
| Year | 2004 |
| PMID | 14985626 |
| Species | Rat |
| Journal | Biol Trace Elem Res |
| RefScore | 2 |
| Source | PArchNLP |
  ||  |  |


---

|  |  |
| --- | --- |
 Reference:3 || Sentence | Reduced dietary iron was found to increase maternal liver copper, inversely correlating with iron levels. |
| Year | 2004 |
| PMID | 15157943 |
| Species | Human |
|  | Rat |
| Journal | J Nutr Biochem |
| RefScore | 2 |
| Source | PArchNLP |
  |


---

|  |  |
| --- | --- |
